# Compartmentalization, a key mechanism controlling the multitasking role of the SnRK1 complex

**DOI:** 10.1093/jxb/erac315

**Published:** 2022-07-21

**Authors:** Emilio Gutierrez-Beltran, Jose L Crespo

**Affiliations:** Instituto de Bioquimica Vegetal y Fotosintesis, Consejo Superior de Investigaciones Cientificas (CSIC)-Universidad de Sevilla, Sevilla, Spain; Departamento de Bioquimica Vegetal y Biologia Molecular, Facultad de Biologia, Universidad de Sevilla, Sevilla, Spain; Instituto de Bioquimica Vegetal y Fotosintesis, Consejo Superior de Investigaciones Cientificas (CSIC)-Universidad de Sevilla, Sevilla, Spain; Instituto Gulbenkian de Ciência, Portugal

**Keywords:** Autophagy, plant cell, SnRK1 compartmentalization, SnRK1-interacting proteins, SnRK1/TOR signaling, stress granules

## Abstract

SNF1-related protein kinase 1 (SnRK1), the plant ortholog of mammalian AMP-activated protein kinase/fungal (yeast) Sucrose Non-Fermenting 1 (AMPK/SNF1), plays a central role in metabolic responses to reduced energy levels in response to nutritional and environmental stresses. SnRK1 functions as a heterotrimeric complex composed of a catalytic α- and regulatory β- and βγ-subunits. SnRK1 is a multitasking protein involved in regulating various cellular functions, including growth, autophagy, stress response, stomatal development, pollen maturation, hormone signaling, and gene expression. However, little is known about the mechanism whereby SnRK1 ensures differential execution of downstream functions. Compartmentalization has been recently proposed as a new key mechanism for regulating SnRK1 signaling in response to stimuli. In this review, we discuss the multitasking role of SnRK1 signaling associated with different subcellular compartments.

## Teaser

This review discusses the SnRK1 signaling associated with different subcellular compartments and how this compartmentalization may contribute to the multitasking role of the SnRK1 complex.

## Introduction

Plants are sessile organisms continuously exposed to a wide range of environmental cues including light, wounding, or temperature, which have a major impact on their development and productivity. Consequently, they have developed sophisticated cellular mechanisms to survive in ever-changing environments. In this regard, the evolutionarily conserved protein SNF1-related kinase 1 (SnRK1) is considered as a master regulator that integrates external signals with plant growth (Baena-Gonzalez *et al.*, 2007; Broeckx *et al.*, 2016). SnRK1 is activated by sugar starvation, promoting the phosphorylation of a large number of proteins (Jamsheer *et al.*, 2021). Arabidopsis SnRK1 and its orthologs, the yeast sucrose non-fermenting-1 protein kinase (SNF1) and mammalian AMP-activated protein kinase (AMPK), operate as a heterotrimeric complex composed of a catalytic α-subunit and two regulatory subunits, β and γ. In plants, a hybrid SnRK1βγ protein (with a carbohydrate-binding domain typically found in β-subunits) functions as the γ-subunit. While the kinase α-subunit is required for activation of signaling events associated with SnRK1, β- and βγ-subunits control SnRK1α activity, localization, and substrate specificity (Jamsheer *et al.*, 2021).

**Fig. 1.**
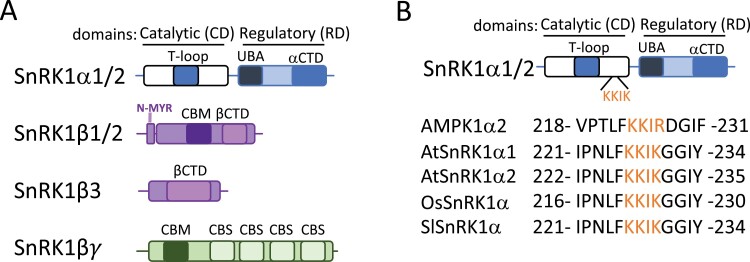
SnRK1 subunit architecture. (A) SnRK1α subunits contain a Ser/Thr kinase domain (referred to as the catalytic domain; CD) at the N-terminus followed by a regulatory domain (referred to as the RD) at the C-terminus. The CD contains an activation loop (T-loop), while the C-terminal part includes both ubiquitin-associated (UBA) and far C-terminal (αCTD) subdomains. The regulatory β-subunits consist of an N-terminal myristoylation (N-MYR) motif, a carbohydrate-binding module (CBM), and a β-C-terminal domain (βCTD) for SnRK1β1 and SnRK1β1, and a βCTD for SnRK1β3. The regulatory βγ-subunit combines four cystathionine-β-synthase (CBS) domains at the C-terminus with an N-terminal CBM. (B) Multiple amino acid sequence alignment of the putative nuclear localization signal (NLS) of SnRK1 from *Arabidopsis thaliana* (AtSnRK1α), *Oryza sativa* (OsSnRK1α), *Solanum lycopersicum* (SlSnRK1α) and human (AMPK1α2). The NLS is marked in orange.

In Arabidopsis, the catalytic α-subunit of SnRK1 is encoded by three genes, SnRK1α1, SnRK1α2, and SnRK1α3 (also referred to as *AKIN10/AKIN11/AKIN12* or *KIN10/ KIN11/KIN12*), of which α1 and α2 are partially redundant (Baena-Gonzalez *et al.*, 2007). SnRK1α3, which is poorly expressed, is often considered to be a pseudogene (Baena-Gonzalez *et al.*, 2007; Le *et al.*, 2011), and SnRK1α3 cloning has not been reported yet. Notably, *snrk1α1*/*snrk1α2* (*snrk1α1/1α2*) double knockout appears to be lethal, supporting the non-functionality of SnRK1α3 (Baena-Gonzalez *et al.*, 2007). The domain architecture of the α-subunit is highly conserved and includes a Ser/Thr kinase domain (also referred to as a catalytic domain; CD) at the N-terminus followed by a regulatory domain (RD) at the C-terminus (Fig. 1A). The CD contains an activation loop (T-loop), with a conserved threonine (Arabidopsis SnRK1α1/α2^T175/176^), whose phosphorylation has been reported to be critical for SnRK1 activity (Baena-Gonzalez *et al.*, 2007; Herzig and Shaw, 2018; Lin and Hardie, 2018). In mammals, the level of T-loop phosphorylation parallels AMPK kinase activity, although this correlation does not seem to be so clear in plants (Emanuelle *et al.*, 2015; Herzig and Shaw, 2018; Lin and Hardie, 2018). Likewise, the C-terminal part of the protein includes both a ubiquitin-associated (UBA) domain and a far C-terminal (αCTD) domain. While UBA was found to be crucial for maintaining the catalytic activity of SnRK1α (Emanuelle *et al.*, 2018), the αCTD is required for the interaction with the β- and γ-subunits (Kleinow *et al.*, 2000).

The Arabidopsis genome encodes three β-subunits, SnRK1β1, SnRK1β2, and SnRK1β3. SnRK1β1 and SnRK1β2 are constituted by an N-terminal myristoylation (N-MYR) motif, a carbohydrate-binding module (CBM), and a β-C-terminal domain (βCTD), whereas SnRK1β3 is formed exclusively by a βCTD (Fig. 1A). Although the three β-subunits are involved in SnRK1 signaling (Emanuelle *et al.*, 2018), a lack of studies in these proteins makes it difficult to establish the specific contribution of each domain and/or isoform to the complex. To date, it has been shown that myristylation of the N-MYR motif controls AtSnRK1α activity and localization (Pierre *et al.*, 2007; Ramon *et al.*, 2019). In contrast to mammals or yeast, plants possess an atypical γ-subunit that combines four cystathionine-β-synthase (CBS) domains at the C-terminus with an N-terminal CBM, usually found in β-subunits (Fig. 1A), which explains why this atypical γ-subunit is referred to as the βγ-subunit in plants. The lethality of the Arabidopsis SnRK1βγ knockout mutant suggests an essential role for this gene in plants (Ramon *et al.*, 2013). The binding of adenine nucleotides (ATP, ADP, or AMP) to AMPKγ has been reported as necessary for AMPK activity (Gowans *et al.*, 2013). Although this regulatory mechanism is absent in plants (Emanuelle *et al.*, 2015), several findings suggest that, similar to AMPKγ, the Arabidopsis βγ-subunit is crucial for SnRK1 signaling. For example, a reduced SnRK1βγ expression correlated well with reduced SnRK1 target gene expression (Ramon *et al.*, 2013). On the other hand, the presence of the βγ-subunit is necessary for the heterotrimeric SnRK1α1βγβ3 complex activity in response to maltose (Ruiz-Gayosso *et al.*, 2018).

SnRK1 has been involved in the regulation of important cellular functions, including growth, autophagy, stress response, stomatal development, pollen maturation, hormone signaling, and gene expression (Li *et al.*, 2017; Han *et al.*, 2020; Jamsheer *et al.*, 2021). However, the mechanism whereby SnRK1 ensures differential execution of downstream functions remains to be determined. One possibility is that response specificity may be achieved by stimulus-specific phosphorylation of target proteins. In fact, two recent and independent phosphoproteomic studies indicated that SnRK1α regulates the phosphorylation state of ~500 proteins (Cho *et al.*, 2016; Nukarinen *et al.*, 2016). Another solution to achieve multitasking within the cellular space is compartmentalization. Indeed, SnRK1 has been localized in the cytoplasm, nucleus, plasma membrane, chloroplast, endoplasmic reticulum (ER), and stress granules (SGs) in response to various physiological inputs (Fragoso *et al.*, 2009; Jamsheer *et al.*, 2018b; Gutierrez-Beltran *et al.*, 2021; Song *et al.*, 2021; Sun *et al.*, 2021). To date, all studies performed on plant SnRK1 have been focused on understanding the mechanistic implication of SnRK1 activation. However, little is known about the spatially defined SnRK1 regulation. In this review, we discuss the SnRK1 signaling associated with different subcellular compartments and how this compartmentalization may contribute to the multitasking role of the SnRK1 complex.

## Linking SnRK1 localization with functional output

### Signal-dependent nuclear shuttling of SnRK1α as a mechanism for controlling gene expression

**Fig. 2.**
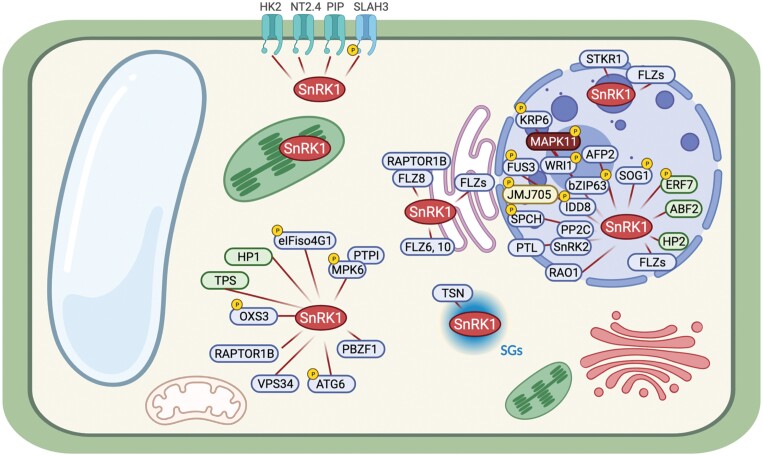
SnRK1 downstream substrates identified in plants. Subcellular localization and interaction data of SnRK1 were retrieved from the literature (see Supplementary Table S1). SnRK1 interaction proteins are marked in blue, green, yellow, or red, when the interaction is reported in *Arabidopsis thaliana*, *Glycine max*, *Oryza sativa*, or *Solanum lycopersicum*, respectively. The phosphorylation targets of SnRK1 are marked with a yellow circle with a P inside. The interactors were annotated using TAIR v10. The figure was created with BioRender.

Numerous studies have demonstrated the presence of the catalytic α-subunit in the nucleus. In fact, a large number of proteins have been reported to interact with SnRK1α in this organelle (Fig. 2; Supplementary Table S1). Although most of these studies are based on transient expression assays in *Nicotiana benthamiana* and Arabidopsis protoplasts, it is well established that SnRK1α shuttles between the cytoplasm and nucleus under certain conditions. For example, low-energy stress triggers a change in localization of AtSnRK1α1 from the cytoplasm to the nucleus (Ramon *et al.*, 2019). More recently, the nuclear interaction between *Oryza sativa* SnRK1α1 and the histone H3K27me3 demethylase JMJ705 was enriched under starvation stress (Wang *et al.*, 2021). However, the mechanism that regulates the cytoplasm to nuclear translocation of SnRK1 is unknown. Studies on mammalian models have revealed that a conserved sequence localized at the N-terminus of AMPKα facilitates the signal-dependent shuttling between the cytoplasm and nucleus (Suzuki *et al.*, 2007; Kazgan *et al.*, 2010). In particular, the amino acid sequence KKIR located in the catalytic domain of AMPKα2 was essential for nuclear translocation in response to the hormone leptin (Suzuki *et al.*, 2007). Considering that the minimum requirement for a monopartite nuclear localization signal (NLS) is Lys-(Lys/Arg)-X-(Lys/Arg) (Lu *et al.*, 2021), an amino acid sequence alignment of SnRK1α proteins from several plant species showed a high conservation of the KKIK sequence (Fig. 1B), suggesting a possible conservation of the mechanism.

The current model for AMPKα action/function indicates that cytoplasmically activated protein is translocated to the nucleus where it promotes phosphorylation of downstream transcriptional regulators to control gene expression (Chauhan *et al.*, 2020). In fact, the phosphorylation of the conserved Thr172 residue is essential for the nuclear translocation of the protein (Suzuki *et al.*, 2007). Similar to the mammalian ortholog, SnRK1α has been found to phosphorylate a large number of transcription regulators in plants, among them Arabidopsis indeterminate domain 8 (AtIDD8), WRINKLED1 (AtWR1), osJMJ705, or *Glycine max* AP2/ERF domain-containing protein (GsERF7) (Fig. 2; Supplementary Table S1). However, whether SnRK1α phosphorylation is required for the nuclear translocation of the protein is currently unknown. To date, a clear correlation between T-loop phosphorylation and nuclear function of SnRK1α has been established based on the following observations: (i) gene expression triggered by SnRK1α is inhibited in plants expressing the inactive mutant form AtSnRK1α1^T175A^ (Baena-Gonzalez *et al.*, 2007; Cho *et al.*, 2012); (ii) SnRK1α-dependent degradation of the transcription factor AtWRI1 does not take place when AtSnRK1α1^T175A^ is expressed (Zhai *et al.*, 2017); (iii) similarly to the SnRK1α wild-type form, the constitutively active form AtSnRK1α1^T175D^ is translocated to the nucleus and promotes stabilization of the transcription factor SPEECHLESS (SPCH) (Han *et al.*, 2020); and (iv) phosphorylation of Thr175 is required for Arabidopsis gene expression (Ramon *et al.*, 2019).

SnRK1α has been recently localized in nuclear bodies (NBs), suggesting an exciting and unexplored role for the complex in the nucleus (Blanco *et al.*, 2019). This localization was also observed by bimolecular fluorescence complementation (BiFC) experiments, in which AtSnRK1α and interacting partners were found to interact in these structures. The AtSnRK1α-interacting proteins include several members of the FCS-like zinc fingers family (FLZs, earlier known as DUF581) and GL1 enhancer-binding protein (GeBP) (Fig. 2; Supplementary Table S1) (Nietzsche *et al.*, 2014, 2018). NBs are biomolecular condensates whose functional role in plants remains largely unknown. However, several recent findings support the model whereby NBs have key roles in nuclear functions in response to environmental stimuli (Meyer, 2020). Abscisic acid (ABA) is a phytohormone essential for plant response to environmental stress that mediates SnRK1 signaling (Rodrigues *et al.*, 2013; Belda-Palazon *et al.*, 2020). Moreover, several SnRK1α-interacting proteins involved in ABA signaling have been reported to localize in NBs, such as ABA-insensitive 5 (ABI5), ABI5-binding protein (AFP), phytochrome-interacting factor 4 (PIF4), or WRKY family members (Lopez-Molina *et al.*, 2003; Geilen and Bohmer, 2015; Hwang *et al.*, 2019; Carianopol *et al.*, 2020). These findings, together with the fact that SnRK1 and ABA signaling were found to regulate a common set of stress-responsive genes (Rodrigues *et al.*, 2013), suggest a role for NB-dependent SnRK1 localization in ABA-mediated regulation of gene expression in plants. However, the biological significance of this localization is yet to be identified.

### The endoplasmic reticulum as a platform for SnRK1/TOR regulation through FLZ proteins

**Fig. 3.**
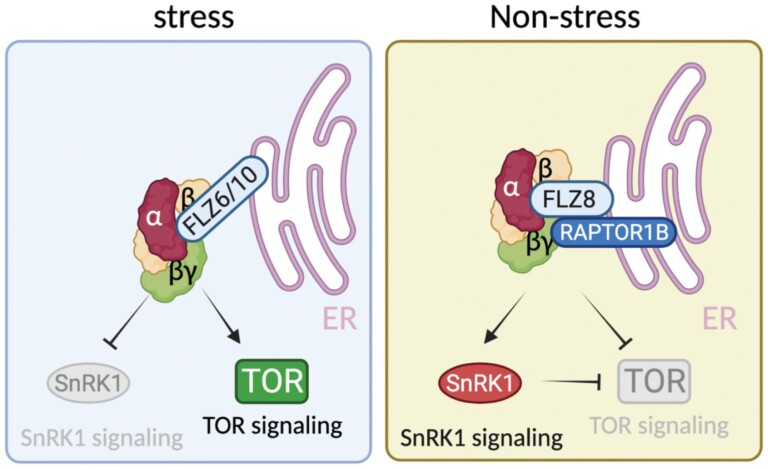
Model for the SnRK1/TOR/FLZ signaling network. Under unfavorable conditions, both FLZ6 and FLZ10 repress SnRK1, allowing TOR signaling, in an ER localization manner. Under favorable conditions, FLZ8 induces TOR signaling inhibition by two different mechanisms, namely (i) promoting SnRK1 signaling through enhancing the SnRK1α1 level and (ii) stimulating RAPTOR1B–SnRK1α1 interaction in the ER. Lines with arrows indicate positive regulation and lines with bars indicate negative regulation. The figure was created with BioRender.

A study using both transient and stable expression in plants has shown that SnRK1α is stably associated with the ER (Blanco *et al.*, 2019). The ER is a dynamic cellular organelle involved in protein synthesis, peptide chain folding, and trafficking (Manghwar and Li, 2022). Apart from its central role in protein synthesis, the ER is also involved in regulating the stress response in plant cells (Liu and Li, 2019). A previous study demonstrated that AtSnRK1α is able to interact with at least 10 members of the FLZ protein family in the ER (Fig. 2; Supplementary Table S1) (Jamsheer *et al.*, 2018a, b). FLZs are small proteins with a C2–C2 FLZ domain that have been involved in the regulation of abiotic stress and ABA responses (He and Gan, 2004; Chen *et al.*, 2013). From the FLZ family, both FLZ6 and FLZ10 were reported first to interfere with the SnRK1/target of rapamycin (TOR) signaling pathways (Jamsheer *et al.*, 2018a). Thus, protein levels of AtSnRK1α were found to be enhanced in *flz6* and *flz10* single mutants, while the level of phosphorylated ribosomal protein S6 kinase (S6K), a well-established target of TOR, was found to be reduced. The authors propose a model where the interaction of SnRK1α with both FLZ6/10 proteins in the ER may mediate the antagonist signaling of the SnRK1/TOR module in plants under unfavorable conditions (Fig. 3).

The relevance of the ER for SnRK1/TOR signaling is also strengthened by the fact that three members of the TOR complex (TORC), namely TOR, regulatory-associated protein of TOR (RAPTOR), and lethal with SEC13 protein 8 (LST8) have been localized in the ER in distant lineages such as animals or algae (Liu and Zheng, 2007; Diaz-Troya *et al.*, 2008; Yadav *et al.*, 2013). In fact, the ER localization of mammalian TOR (mTOR) has been reported to be crucial for its activity (Liu and Zheng, 2007). Jamsheer *et al.* (2022) have recently suggested that FLZ8, another member of the FLZ family, may act as a scaffold protein regulating SnRK1/TOR activity in plants. They found that FLZ8 negatively regulates TOR signaling by two different mechanisms: (i) stimulating antagonistic SnRK1α1 signaling and (ii) promoting SnRK1α1/RAPTOR1B association (Fig. 3). Notably, the FLZ8–SnRK1α–RAPTOR1B association was found to take place in the ER (Fig. 2; Supplementary Table S1). Interestingly, the potential role of FLZ family proteins as scaffolds has been recently highlighted in a new study (Bortlik *et al.*, 2022, Preprint). In this work, the authors found that FLZ3 inhibits SnRK1 activity by interfering with the upstream activating kinase GRIK2. Moreover, FLZ3 was found to localize in the ER (Jamsheer *et al.*, 2018b). Collectively, these studies suggest a possible role for the ER as a hub for SnRK1/TOR regulation mediated by FLZ proteins in plants, although further studies are needed to demonstrate the specific contribution of individual FLZ proteins to the regulation of SnRK1/TOR signaling.

### Chloroplasts, a hub for SnRK1-mediated starch metabolism regulation?

Both Arabidopsis SnRK1α1 and SnRK1α2 isoforms were found to be localized inside and around the chloroplast (Fragoso *et al.*, 2009; Ruiz-Gayosso *et al.*, 2018; Blanco *et al.*, 2019). Although the functionality of this localization is still an open question, several studies indicate the existence of a convincing link between SnRK1α signaling and the organelle. A quantitative phosphoproteomic study indicated that the phosphorylation status of several proteins with a known role in chloroplast light reactions was down-regulated in the *snrk1α1/1α2* double mutant compared with wild-type plants (Nukarinen *et al.*, 2016). Further works using protein–protein interaction approximations found a clear link between SnRK1 and chloroplast function and development (Rohila *et al.*, 2009; Carianopol *et al.*, 2020). A recent study showed that treatment with DCMU [3-(3,4-dichlorophenyl)-1,1-dimethylurea], a known inhibitor of chloroplast electron transport, causes a profound effect on SnRK1α localization, showing a re-localization from the non-nuclear to the nuclear fraction (Blanco *et al.*, 2019). Accordingly, activation of AtSnRK1 kinase activity has been reported under energy deprivation triggered by both DCMU treatment and prolonged darkness (Baena-Gonzalez *et al.*, 2007; Kim *et al.*, 2017). The latter scenario is known to promote degradation of chloroplast proteins and chlorophyll, leading to a misregulation of the chloroplast function and an imbalance in the cellular redox state (Dietz *et al.*, 2016; Kim *et al.*, 2017). In close agreement, SnRK1α activity has been recently reported to be strongly dependent on the redox state (Wurzinger *et al.*, 2017). All these results point to a functional connection of SnRK1 activity with the chloroplast, but whether it is direct or indirect is unknown.

Besides SnRK1α isoforms, the regulatory β- and βγ-subunits have been localized in the chloroplast (Fragoso *et al.*, 2009; Avila-Castaneda *et al.*, 2014; Ruiz-Gayosso *et al.*, 2018). Among them, SnRK1β1, SnRK1β2, and SnRK1βγ share a CBM domain (Fig. 1A), a domain known to inhibit AMPK activity when bound to glycogen (Koay *et al.*, 2010). Starch, the plant analog of glycogen, is stored inside the chloroplast as a transitory polysaccharide granule. Initially, the CBMs from SnRK1β2 and SnRK1βγ were described to bind starch *in vitro* (Avila-Castaneda *et al.*, 2014). However, a later study using AMPKβ subunits as positive controls reported that SnRK1 CBMs cannot bind to this polysaccharide (Emanuelle *et al.*, 2015). A subsequent study reported that maltose, the main product of starch degradation at night, binds to SnRK1β1 and SnRK1β2 subunits, and to the SnRK1βγ/β3 complex *in vitro* (Ruiz-Gayosso *et al.*, 2018). Given that the SnRK1β3 subunit lacks a CBM domain, its capacity to bind maltose might be facilitated by forming a complex with the βγ-subunit (Ruiz-Gayosso *et al.*, 2018). Curiously, when the impact of maltose binding on SnRK1 activity was analyzed, only the complex formed by α1/β3/βγ was stimulated, indicating a possible level of control depending on which subunit is assembled. Based on these results, Ruiz-Gayoss *et al.* proposed a model in which the accumulation of maltose at night promotes the increase of SnRK1 activity, inducing maltose metabolism via an as yet undefined mechanism. This finding, together with other studies, suggests that the SnRK1 complex might promote the carbon flux from starch to degradation products (Thelander *et al.*, 2004; Baena-Gonzalez *et al.*, 2007). However, although these findings suggest a possible role for the SnRK1 complex in starch metabolism, both maltose binding and chloroplast localization should be further confirmed.

### The cytoplasm, a meeting place for SnRK1/TOR signaling, stress granules, and autophagy

The cytoplasm is the major intracellular fluid where a plethora of important biological reactions take place. As part of the cytosolic pool, SnRK1 occupies a key position involved in numerous reactions that include involvement in protein synthesis and degradation or stress response signaling. As an example of the latter, SnRK1α has been reported to interact in the cytosol with proteins involved in both biotic and abiotic stress responses (Cho *et al.*, 2016; Chen *et al.*, 2021; Gutierrez-Beltran *et al.*, 2021). For instance, SnRK1α was found to interact with MPK6 and its regulator [protein tyrosine phosphatase 1 (PTP1)] in the cytoplasm under hypoxia caused by submergence (Fig. 2; Supplementary Table S1) (Bartels *et al.*, 2009). This observation suggested the existence of an SnRK1α–PTI1–MPK6 cascade during submergence, which was later confirmed by Cho *et al.* (2016). This study found that SnRK1-induced phosphorylation of PTP1 disrupted the PTP1–MPK6 association, promoting the activation of nuclear target genes dependent on MPK6. On the other hand, IFiso4G1 and eIFiso4G2, two translation initiation factors, were also reported to be cytosolic partners of SnRK1α1 during submergence (Cho *et al.*, 2019). The phosphorylation of both translation initiation factors via SnRK1α1 promoted the cytosolic translation of core hypoxia and stress response genes during submergence.

The assembly of SGs takes place in the cytosol. SGs are cytoplasmic biomolecular condensates that assemble transiently in response to both environmental and internal signals as an adaptive survival mechanism (Alberti and Carra, 2018; Hofmann *et al.*, 2021). SGs typically contain translationally arrested mRNAs, small ribosomal subunits, various translation initiation factors (eIFs), poly(A)-binding proteins (PABs), and a variety of RNA-binding proteins and non-RNA-binding proteins (Protter and Parker, 2016). SnRK1α has been recently reported to be among the multiple proteins associated with SGs, and both SnRK1α1 and SnRK1α2 isoforms from Arabidopsis have been shown to interact with Tudor staphylococcal nuclease (TSN) in these membraneless organelles (Gutierrez-Beltran *et al.*, 2021). TSN is a scaffold protein required for the proper assembly of plant SGs (Gutierrez-Beltran *et al.*, 2016). The formation of SGs and the presence of TSN are required for the activation of SnRK1 signaling in response to heat stress (Gutierrez-Beltran *et al.*, 2021). Although the link between SnRK1 and plant SG assembly is still poorly understood, the role of their yeast and animal homologs in SG biogenesis is well known. Thereby, the presence of AMPKα or SNF1 is required for the proper assembly of SGs in largely divergent organisms such as *Caenorhabditis elegans*, *Saccharomyces cerevisiae*, or mammals (Hofmann *et al.*, 2012; Mahboubi *et al.*, 2015a; Kuo *et al.*, 2020). Furthermore, the pharmacological activation of AMPKα affects key aspects of SG biology, including assembly and fusion (Mahboubi *et al.*, 2015a, 2016). In this respect, both β and γ regulatory subunits have been also localized in SGs in mammalian cells (Mahboubi *et al.*, 2015b). Given the pro-survival role of SGs, the effect of SnRK1/AMPK/SNF1 on SG biogenesis may be considered as a new avenue for modulating cell survival in response to stress.

**Fig. 4.**
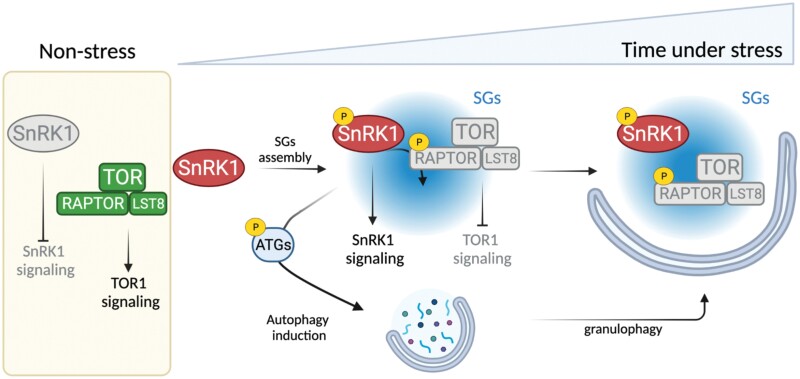
Hypothetical model for SG-dependent regulation of the SnRK1/TOR signaling network. Under favorable conditions, SnRK1 activity is repressed while TOR signaling is activated, promoting processes associated with cell proliferation and growth. Following stress perception, sequestration and activation of SnRK1 in SGs might contribute to TOR signaling inhibition by SnRK1-dependent phosphorylation of RAPTOR1B. At the same time, SG-dependent SnRK1 activation induces stress responses, which might include activation of autophagy (via ATG phosphorylation). Finally, autophagy might mediate SG degradation via granulophagy. Lines with arrows indicate positive regulation and lines with bars indicate negative regulation. The figure was created with BioRender.

A previous study reported that Arabidopsis SnRK1α1 phosphorylates (*in vitro*) and interacts with RAPTOR1B in the cytoplasm (Nukarinen *et al.*, 2016). RAPTOR1B is part of the TORC in plants, which also includes LST8. The Arabidopsis genome contains two copies for *RAPTOR* (*RAPTOR1A* and *RAPTOR1B*) and *LST8* (*LST8-1* and *LST8-2*) genes, although *LST8-2* shows undetectable transcript levels (Anderson *et al.*, 2005; Moreau *et al.*, 2012). In contrast to plants, mammalian cells contain two different TOR complexes, mTORC1 (homolog to plant TORC) and mTORC2. The latter is formed by the association of rapamycin-insensitive companion of TOR (RICTOR) and mammalian stress-activated protein kinase-interacting protein 1 (mSIN1). In yeast and mammalian models, AMPK/SNF1 are well-established upstream negative regulators of TORC1. While phosphorylation of mammalian RAPTOR (mRAPTOR) via AMPKα promotes the inhibition of mTOR kinase activity (Gwinn *et al.*, 2008), this link is not so obvious in yeast (Hughes Hallett *et al.*, 2015). In Arabidopsis, a recent study revealed that the cytoplasmic interaction between SnRK1α1 and TOR is required for TOR inhibition in response to stress (Belda-Palazon *et al.*, 2020,  2022). However, whether this control is mediated by RAPTOR is still an open question. Together with a previous study showing TOR inhibition by stress-induced phosphorylation of RAPTOR1B in Arabidopsis by SnRK2 (Wang *et al.*, 2018), these findings strongly suggest the existence of a SnRK1–RAPTOR–TOR regulatory network in plants. In this respect, SGs might operate as a platform for this signaling module in plants. Both RAPTOR and mTOR are bona fide SG components in the mammalian system (Rehbein *et al.*, 2021). Growing evidence indicates that SGs constitute a cytoplasmic compartment in which mTORC1 is inhibited under stress through several mechanisms, which include sequestration of both RAPTOR and mTOR proteins (Thedieck *et al.*, 2013; Wippich *et al.*, 2013; Mediani *et al.*, 2021; Prentzell *et al.*, 2021). Given that stress-induced localization of SnRK1α in SGs promotes its activation (Gutierrez-Beltran *et al.*, 2021), one possibility is that SnRK1α regulates TOR signaling inhibition by phosphorylation of SG-localized RAPTOR1B (Fig. 4). It is very well established that TOR acts as a central metabolic regulator playing largely antagonistic roles to SnRK1 (Margalha *et al.*, 2019). Therefore, the association of SnRK1α–TOR with SGs may imply a checkpoint for the activation/inhibition of these signaling pathways which fully depends on the cellular homeostasis (see Fig. 4 for a hypothetical model).

It is well known that AMPK and TOR antagonistically regulate autophagy in mammalian cells (Gonzalez *et al.*, 2020). While TOR has been postulated to act as a negative regulator, AMPK plays a positive role in autophagy dynamics. In contrast to the mammalian model, the molecular mechanism of SnRK1/TOR-mediated control of autophagy in plants is still under study. Several recent findings point to the presence of a common nexus between the SnRK1/TOR module and autophagy via autophagy-related (ATG) proteins (Liao and Bassham, 2020). Hence, the phosphorylation of Arabidopsis ATG13 is considered as a key modification whereby TOR mediates the inhibition of autophagy (Son *et al.*, 2018). Regarding SnRK1, a recent study has shown that the phosphorylation and interaction with ATG6 in cytoplasmic foci promotes autophagy during prolonged carbon starvation in Arabidopsis (Fig. 2; Supplementary Table S1) (Huang *et al.*, 2019). Previously, it was demonstrated that overexpression of AtSnRK1α1 enhanced both autophagosome formation and ATG1a phosphorylation *in vivo* (Chen *et al.*, 2017). Among ATG proteins, ATG1, ATG13, or ATG6 are required for earlier events of autophagy induction, suggesting that the SnRK1/TOR module might act early in autophagy regulation (Huang *et al.*, 2019). Based on the fact that mammalian AMPK controls both autophagy induction and SG dynamics, we hypothesize that SG-localized SnRK1 may be involved in autophagy activation via phosphorylation of key ATG proteins (Fig. 4). In mammals, autophagy controls SG disassembly through a process known as granulophagy (Seguin *et al.*, 2014; Hofmann *et al.*, 2021). Indeed, ULK1 and ULK2 proteins, the mammalian orthologs of ATG1, have been shown to promote SG disassembly (Wang *et al.*, 2019). In plants, granulophagy has been described to control SG disassembly during extended hypoxia (Field *et al.*, 2021, Preprint). However, whether activation of autophagy under stress-induced SG assembly conditions as well as granulophagy is controlled by the SnRK1/TOR module is totally unknown.

### The plasma membrane links SnRK1 and channel regulation

Arabidopsis SnRK1α1 interacts with the cytosolic C-terminal region of the plasma membrane (PM) protein SLAC1 homolog 3 (SLAH3) (Fig. 2; Supplementary Table S1) (Sun *et al.*, 2021). SLAH3 is an anion channel involved in the efflux of NO_3_^–^ under high-NH_4_^+^/low- NO_3_^–^ conditions as a mechanism of ammonium detoxicity in plants (Zheng *et al.*, 2015). It has been proposed that under physiological growth conditions, the cytosol-localized AtSnRK1α1 interacts with and phosphorylates SLAH3 to inhibit its activity, preventing nitrate loss (Zheng *et al.*, 2015). When the concentration of NH_4_^+^ is high, active AtSnRK1α1 migrates to the nucleus, which releases the inhibition of SLAH3 and leads to nitrate efflux. This is the first evidence showing the regulation of channel activity via SnRK1 in plants. However, previous studies identified other channels or transporters as SnRK1 interactor partners. For example, a yeast two-hybrid (Y2H) assay showed that both aquaporin PIP1 and nitrate transporter 2.4 (NT2.4) interact with soybean SnRK1α (Song *et al.*, 2019). More recently, protein–protein interaction analysis using a Y2H assay revealed the interaction of AtSnRK1α with several cyclic nucleotide-gated channels (CNGCs), including CNGC12, CNGC13, and CNGC18, as well as channels involved in phosphate transport such as phosphate transporter 1;4 (PHT1; 4) and PHO1 homolog 7 (PHO1; H7) (Carianopol *et al.*, 2020; Jamsheer *et al.*, 2021).

Similar to plants, several studies in mammals have shown that AMPK directly or indirectly alters the activities of various channels (Lang and Foller, 2014). For example, AMPKα phosphorylates and inhibits BK_Ca_, a voltage-gated potassium channel (Wyatt *et al.*, 2007). Furthermore, AMPKα also controls the channel activity via intermediates. For example, AMPKα stimulates Nedd4.2, a ubiquitin ligase that mediates the down-regulation of the epithelial Na^+^ channel ENaC (Bhalla *et al.*, 2006). However, it remains unknown whether this undirect mechanism of regulation exists in plants. In both plants and mammalian models, the interaction between AMPK/SnRK1 and channels was detected at the PM (Fig. 2; Supplementary Table S1) (Lang and Foller, 2014; Sun *et al.*, 2021). Membrane localization of the AMPK/SnRK1 complex has been reported to be controlled by N-terminal myristoylation of the regulatory β-subunits (Lin *et al.*, 2003; Oakhill *et al.*, 2010; Ramon *et al.*, 2019). One possibility is that the phosphorylation/interaction of SnRK1 with channels is mediated via myristoylation, although this has not been explored. Taken together, these studies suggest that control of channel activity via SnRK1 may be a common feature in plants. However, a thorough analysis should be performed to demonstrate the biological significance of these interactions.

### Is the plant vacuole a key hub for SnRK1/TOR signaling?

The lysosome (or vacuole in yeast and plants) is a membrane-bound organelle that facilitates the digestion of macromolecules. However, lysosomes have been also proposed to have a key role in other cellular processes including cellular differentiation, metabolism, or signaling regulation (Lim and Zoncu, 2016; Young *et al.*, 2016; Abu-Remaileh *et al.*, 2017). Moreover, several studies have linked lysosomes as a hub for the mechanistic regulation of AMPK/mTOR via a v-ATPase-Ragulator complex (Carroll and Dunlop, 2017). Thus, under glucose starvation, the v-ATPase promotes assembly of an AXIN–liver kinase B1 (LKB1) complex at the lysosome surface to activate AMPK (Zhang *et al.*, 2014). At the same time, v-ATPase facilitates the release of mTORC1 from the lysosome surface, leading to the inhibition of mTORC1 activity (Zhang *et al.*, 2014). A growing body of evidence indicates now that SGs might also be involved in the lysosomal regulation of mTORC1 activity via the core SG marker G3BP1 (Rehbein *et al.*, 2021). Indeed, G3BP1 has been reported to anchor the tuberous sclerosis complex (TSC) to lysosomes and suppress mTORC1 signaling (Prentzell *et al.*, 2021). In the budding yeast, glucose starvation has been reported to increase the threshold for TORC1 activation when Kog1/RAPTOR is re-localized from the vacuolar membrane to a single body near the edge of the organelle, in an event dependent on SNF1 (Hughes Hallett *et al.*, 2015). These findings reveal a key hub role for the lysosome/vacuole organelle in AMPK/TOR regulation. Whether such mechanisms of regulation via the vacuole exist in plants requires further investigation.

## Factors affecting the subcellular localization of SnRK1

### N-Terminal myristoylation is a key process controlling SnRK1 localization

The Arabidopsis SnRK1β1 and SnRK1β2 subunits, but not SnRK1β3, have an N-MYR motif and are myristoylated *in vivo* on a conserved glycine residue at position 2 (Gly2) (Fig. 1A). *N*-myristoylation is catalyzed by *N*-myristoyltransferase (NMT) and consists of the addition of the 14-carbon fatty acid, myristate, to the N-terminus via a covalent amide bond. This post-translational modification facilitates the association of proteins with cellular membranes. In the case of SnRK1, *N*-myristoylation of β-subunits has been reported to control both SnRK1 localization and activity (Lin *et al.*, 2003; Pierre *et al.*, 2007; Oakhill *et al.*, 2010; Broeckx *et al.*, 2016; Ramon *et al.*, 2019). Thus, the *N*-myristoylation of both regulatory β-subunits has been reported to negatively regulate the nuclear translocation of SnRK1α1, whose localization is required for SnRK1-induced target gene activation during metabolic stress (Ramon *et al.*, 2019). A previous work found that loss of NMT activity leads to an enhancement of SnRK1-associated kinase activity, providing evidence of *N*-myristoylation-dependent activation of SnRK1 (Pierre *et al.*, 2007). However, whether this phenotype is caused by a defect in SnRK1β-dependent recruitment of the α-subunit to membranes is still an open question. In mammals, N-terminal myristoylation of the β-subunits has been shown to suppress AMPKα activity, keeping AMPKα in an inactive state at the membrane (Warden *et al.*, 2001; Oakhill *et al.*, 2010). Nevertheless, the mechanism whereby N-terminal myristoylation mediates SnRK1 activity is still under study.

### The subcellular localization of SnRK1 changes in a stimulus-dependent manner

As discussed above, the SnRK1α subunit is localized at the cytoplasm, nucleus, chloroplast, ER, or SGs, and this localization seems to be stimulus dependent in some cases. For example, under non-stress conditions, the α1 isoform exhibits a nuclear localization that is particularly prominent in Arabidopsis root meristem cells, and it delocalizes to the cytoplasm in response to ABA (Belda-Palazón *et al.*, 2022). This phenomenon appears to be required for the cytoplasmic control of TOR activity in response to ABA (Belda-Palazon *et al.*, 2020, 2022). The cytoplasm to nucleus migration of SnRK1α1 has been described as a mechanism to induce, but not repress, target gene expression under metabolic, hypoxia, DCMU, dark, or high-ammonium stresses (Ramon *et al.*, 2019; Sun *et al.*, 2021; Wang *et al.*, 2021). In another work, the localization in SGs of both α1 and α2 isoforms was described to be heat stress dependent (Gutierrez-Beltran *et al.*, 2021). Notably, the heat-induced SG localization was linked with both T-loop activation and gene expression. Apart from the stress type, the degree of the stress has been also found to generate a response in the compartmentalized pools of AMPK. Thus, a recent study in mouse embryonic fibroblasts (MEFs) reported that compartmentalized AMPKs undergo a hierarchical activation, which fully depend on the intensity of the stress (Zong *et al.*, 2019). Whether this level of regulation exists in plants is completely unknown. In contrast to plants, the activity of compartmentalized AMPK pools has been extensively studied. For instance, the design of biosensors has largely contributed to monitor the spatiotemporal activation of AMPK across multiple organelles in response to stress (Tsou *et al.*, 2011; Miyamoto *et al.*, 2015).

### Differential SnRK1 heterotrimeric complex assembly and expression as a level of specificity

**Fig. 5.**
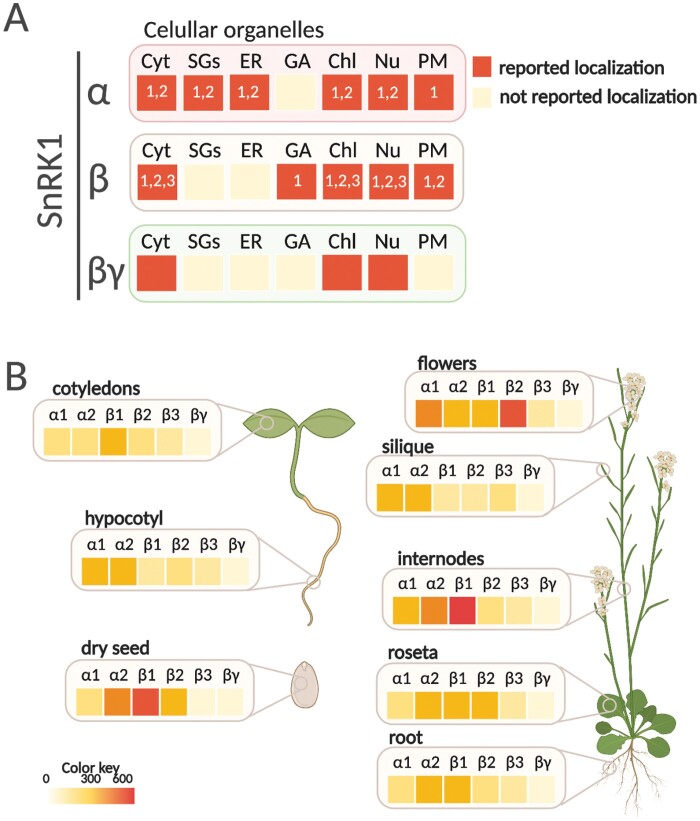
Subcellular localization and expression pattern of SnRK1 subunits. (A) SnRK1 subunits have been visualized in the cytoplasm (Cyt), stress granules (SGs), endoplasmic reticulum (ER), Golgi (AG), chloroplast (Chl), nucleus (Nu), and plasma membrane (PM) in plants. The numbering inside the square indicates the type of subunit. Although subcellular localization of SnRK1 subunits has been retrieved from the literature (see Supplementary Table S2), some of them are controversial and should be corroborated by additional studies (e.g. chloroplast localization of SnRK1α). (B) *In silico* analysis of SnRK1 subunit gene expression using Tissue Specific Root eFP tool (http://bar.utoronto.ca/eplant/). The figure was created with BioRender.

A protein complex can play multiple roles by changing the members of its modular constituents. In the case of Arabidopsis SnRK1, the heterotrimeric complex is composed of α-, β- and βγ-subunits, with α and β having different isoforms (Broeckx *et al.*, 2016). According to this protein composition, six different heterotrimeric complexes are possible. In fact, a previous study showed that all six combinations are assembled *in vitro* (Emanuelle *et al.*, 2015), although it is not clear whether all combinations exist *in vivo*. For example, the differential localization described for each of the subunits make some combinations impossible (Fig. 5A; Supplementary Table S2). Thus, under physiological conditions, SnRK1α isoforms, SnRK1β3 and SnRK1βγ, are predominantly localized to the cytoplasm and nucleus, while both SnRK1β1 and SnRK1β2 are limited to the cytoplasm (Gissot *et al.*, 2006; Bitrian *et al.*, 2011; Gao *et al.*, 2016; Ramon *et al.*, 2019). A more recent study, indeed, shows that SnRK1β1 is localized in the Golgi under transient expression in *N. benthamiana* epidermal cells (Fig. 5A) (Wang *et al.*, 2020). A higher level of complexity is observed based on the tissue- or cell type-specific and subcellular localization. For example, under physiological conditions, α1- and βγ-subunits show preferential nuclear localization in Arabidopsis meristematic cells (Bitrian *et al.*, 2011; Belda-Palazón *et al.*, 2022). Similarly, SnRK1α1 is mainly localized in the nucleus in both guard and stomatal Arabidopsis cells (Han *et al.*, 2020). In stigmata and pistils of young flowers, SnRK1α1 accumulated in the cytoplasm, whereas SnRK1βγ is detected predominantly in nuclei (Bitrian *et al.*, 2011; Gao *et al.*, 2016). Notably, it should not be ruled out that the stress signal may trigger a localization change, thus allowing a differential heterotrimeric assembly. In this respect, yeast α- and all three β-subunits change from cytoplasm to specific cellular compartments when glucose becomes limiting (Hedbacker and Carlson, 2008).

The limited studies in plants make it difficult to establish which complexes are assembled *in vivo*. As an exception, a previous study showed the interaction between AtSnRK1βγ and both AtSnRK1β2 and AtSnRK1β3 subunits in the cytoplasm and nucleus when these proteins are overexpressed (Gissot *et al.*, 2006). In mammals, structures for several functional AMPK heterotrimers have been resolved, including α1β1γ1, α1β2γ1, α2β1γ1, and α2β2γ1 (Xiao *et al.*, 2013; Calabrese *et al.*, 2014; Li *et al.*, 2015; Ngoei *et al.*, 2018). In plants, few types of complexes have been proposed to be functional. One of them was SnRK1α1βγβ3, whose kinase activity was enhanced *in vitro* in the presence of maltose compared with α1βγβ1 and α1βγβ1 (Maya-Bernal *et al.*, 2017; Ruiz-Gayosso *et al.*, 2018). Further studies are required to identify downstream targets specific to one particular heterotrimeric combination and isoform-specific effects on SnRK1 function in plants.

Several studies performed in mammals indicated that the gene expression pattern of AMPK subunits vary across tissues and cells, introducing a new level of heterotrimeric assembly complexity (Trefts and Shaw, 2021). Although a more exhaustive analysis of SnRK1 is required, a similar pattern has been observed in plants. For example, a β-glucuronidase (GUS) promoter analysis found that SnRK1β3 is preferably expressed in developing pollen, ovules, and seeds, while β1- and β2-subunits are ubiquitously expressed (Polge *et al.*, 2008). A more recent study using the same approach showed that the expression pattern of SnRK1α2 was more restricted than that of SnRK1α1, whose expression was detected almost ubiquitously in the full plant (Williams *et al.*, 2014). An *in silico* analysis of SnRK1 subunit expression using Tissue Specific Root eFP (http://bar.utoronto.ca/eplant/) revealed that both catalytic α-subunits and the regulatory SnRK1β1 are expressed throughout development and in different tissues (Fig. 5B). Regarding the regulatory subunits, SnRK1β2 showed a high level of expression in flowers, while the expression level of SnRK1β3 was moderate compared with the rest of the subunits (Fig. 5B). This scenario is presumably different when the expression pattern of SnRK1 subunits is analyzed under different stimuli (Baena-Gonzalez *et al.*, 2007; Polge *et al.*, 2008; Williams *et al.*, 2014).

## Conclusions

SnRK1 signaling is an extremely complex pathway that remains poorly understood in plants. Extensive studies have provided resolution of SnRK1 signal transduction under a set of cellular and environmental cues. These studies include the identification of a plethora of phosphorylated and interacting targets. However, it remains largely unknown how SnRK1 can release a stimulus-type-specific response. This review provides a survey on how localized protein interaction can invoke targeted signaling programs. SnRK1 has been found to interact with targets in such different organelles as the cytoplasm, nucleus, chloroplast, ER, and SGs. Moreover, differential heterotrimeric assembly and the subunit expression pattern can add an extra level of specificity in the downstream SnRK1 response. Although several recent works have provided new insights into cellular compartmentalization of SnRK1, further studies are required to unravel the interplay between the spatiotemporal SnRK1 localization and its downstream signaling mechanisms.

## Supplementary data

The following supplementary data are available at *JXB* online.

Table S1. SnRK1-interacting proteins shown in Fig. 1.

Table S2. Subcellular localization of SnRK1 subunits shown in Fig. 5A.

erac315_suppl_Supplementary_Material
