# Supplementary material for: Compartmentalization, a key mechanism controlling the multitasking role of the SnRK1 complex
Source: J Exp Bot. 2022 Jul 21;73(20):7055–67. doi: 10.1093/jxb/erac315 (PMC9664234; doi:10.1093/jxb/erac315)
Supplement: erac315_suppl_Supplementary_Material [file erac315_suppl_supplementary_material.pdf]

Subcellular localization	SnRK1 interactor	SnRK1 subunit	References
Nucleus	FUS3	SnRK1α1	(Tsai and Gazzarrini, 2012)
Nucleus	RAO1	SnRK1α1	(Ng et al., 2013)
Nucleus	KRP6	SnRK1α1	(Guerinier et al., 2013)
Nucleus	FLZs	SnRK1α1	(Nietzsche et al., 2014)
Nucleus	bZIP63	SnRK1α1	(Mair et al., 2015)
Nucleus	PTL	SnRK1α1	(O'Brien et al., 2015)
Nucleus	IDD8	SnRK1α1, SnRK1α2	(Jeong et al., 2015)
Nucleus	WRI1	SnRK1α1, SnRK1α2	(Zhai et al., 2017)
Nucleus	ABF2, HP2	SnRK1α	(Song et al., 2019)
Nucleus	SOG1	SnRK1α1, SnRK1α2	(Hamasaki et al., 2019)
Nucleus	ERF7	SnRK1α	(Feng et al., 2020)
Nucleus	AFP2	SnRK1α1	(Carianopol and Gazzarrini, 2020)
Nucleus	SPCH	SnRK1α1	(Han et al., 2020)
Nucleus	SnRK2.2, PP2CA	SnRK1α	(Belda-Palazon et al., 2020)
Nucleus	JMJ705	SnRK1α1	(Wang et al., 2021)
Nucleus	MAPK11	SnRK1α	(Song et al., 2021)
Nuclear body	FLZs	SnRK1α1	(Nietzsche et al., 2014)
Nuclear body	STKR1	SnRK1α1, SnRK1α2	(Nietzsche et al., 2018)
Cytoplasm	MPK6, PTP1	SnRK1α1	(Cho et al., 2016)
Cytoplasm	RAPTOR1B	SnRK1α1	(Nukarinen et al., 2016)
Cytoplasm	ATG6, VPS34	SnRK1α1, SnRK1α2	(Huang et al., 2019)
Cytoplasm	eIFiso4G-1, -2	SnRK1α1	(Cho et al., 2019)
Cytoplasm	TPS, HP1	SnRK1α	(Song et al., 2019)
Cytoplasm	PBZF1	SnRK1α	(Xiao et al., 2020)
Cytoplasm	OXS3	SnRK1α1	(Chen et al., 2021)
ER	FLZ6, FLZ10	SnRK1α1, SnRK1α2	(Jamsheer *et al.*, 2018a)
ER	FLZs	SnRK1α1, SnRK1α2	(Jamsheer *et al.*, 2018b)
ER	FLZ8	SnRK1α1	(Jamsheer *et al.*, 2022)
PM	*NT2.4, PIP, HK2*	SnRK1α	(Song *et al.*, 2019)
PM	*SLAH3*	SnRK1α1	(Sun *et al.*, 2021)
SGs	TSN1, TSN2	SnRK1α1, SnRK1α2	(Gutierrez-Beltran *et al.*, 2021)

Supplementary table S1. SnRK1 interacting proteins shown in Fig. 1.

SnRK1 subunit	SnRK1 localization	References
SnRK1α1	Cytoplasm	(Ramon *et al.*, 2019)
SnRK1α1	SGs	(Gutierrez-Beltran *et al.*, 2021)
SnRK1α1	ER	(Blanco *et al.*, 2019)
SnRK1α1	Chloroplast	(Fragoso *et al.*, 2009)
SnRK1α1	Nucleus	(Ramon *et al.*, 2019)
SnRK1α1	PM	(Sun *et al.*, 2021)
SnRK1α2	Cytoplasm	(Williams *et al.*, 2014)
SnRK1α2	SGs	(Gutierrez-Beltran *et al.*, 2021)
SnRK1α2	ER	(Jamsheer *et al.*, 2018a)
SnRK1α2	Chloroplast	(Fragoso *et al.*, 2009)
SnRK1α2	Nucleus	(Williams *et al.*, 2014)
SnRK1β1	Cytoplasm	(Ramon *et al.*, 2019)
SnRK1β1	GA	(Wang *et al.*, 2020)
SnRK1β1	Chloroplast	(Ruiz-Gayosso *et al.*, 2018)
SnRK1β1	Nucleus	(Wang *et al.*, 2020)
SnRK1β1	PM	(Pierre *et al.*, 2007)
SnRK1β2	Cytoplasm	(Ramon *et al.*, 2019)
SnRK1β2	Chloroplast	(Ruiz-Gayosso *et al.*, 2018)
SnRK1β2	Nucleus	(Gissot *et al.*, 2006)
SnRK1β2	PM	(Pierre *et al.*, 2007)
SnRK1β3	Cytoplasm	(Ruiz-Gayosso *et al.*, 2018)
SnRK1β3	Chloroplast	(Ruiz-Gayosso *et al.*, 2018)
SnRK1β3	Nucleus	(Gissot *et al.*, 2006)
SnRK1βγ	Cytoplasm	(Ruiz-Gayosso *et al.*, 2018)
SnRK1βγ	Chloroplast	(Ruiz-Gayosso *et al.*, 2018)
SnRK1βγ	Nucleus	(Bitrian *et al.*, 2011)

Supplementary table S2. Subcellular localization of SnRK1 subunits shown in Fig. 5A.

References

Belda-Palazon B, Adamo M, Valerio C, Ferreira LJ, Confraria A, Reis-Barata D, Rodrigues A, Meyer C, Rodriguez PL, Baena-Gonzalez E. 2020. A dual function of SnRK2 kinases in the regulation of SnRK1 and plant growth. Nature Plants **6**, 1345-1353.

Bitrian M, Roodbarkelari F, Horvath M, Koncz C. 2011. BAC-recombineering for studying plant gene regulation: developmental control and cellular localization of SnRK1 kinase subunits. Plant Journal **65**, 829-842.

Blanco NE, Liebsch D, Guinea Diaz M, Strand A, Whelan J. 2019. Dual and dynamic intracellular localization of Arabidopsis thaliana SnRK1.1. Journal of Experimental Botany **70**, 2325-2338.

Carianopol CS, Gazzarrini S. 2020. SnRK1alpha1 Antagonizes Cell Death Induced by Transient Overexpression of Arabidopsis thaliana ABI5 Binding Protein 2 (AFP2). Frontiers in Plant Science **11**, 582208.

Chen W, Li Y, Yan R, Ren L, Liu F, Zeng L, Sun S, Yang H, Chen K, Xu L, Liu L, Fang X, Liu S. 2021. SnRK1.1-mediated resistance of Arabidopsis thaliana to clubroot disease is inhibited by the novel Plasmodiophora brassicae effector PBZF1. Molecular Plant Pathology **22**, 1057-1069.

Cho HY, Lu MJ, Shih MC. 2019. The SnRK1-eIFiso4G1 signaling relay regulates the translation of specific mRNAs in Arabidopsis under submergence. New Phytologist **222**, 366-381.

Cho HY, Wen TN, Wang YT, Shih MC. 2016. Quantitative phosphoproteomics of protein kinase SnRK1 regulated protein phosphorylation in Arabidopsis under submergence. Journal of Experimental Botany **67**, 2745-2760.

Feng X, Feng P, Yu H, Yu X, Sun Q, Liu S, Minh TN, Chen J, Wang D, Zhang Q, Cao L, Zhou C, Li Q, Xiao J, Zhong S, Wang A, Wang L, Pan H, Ding X. 2020. GsSnRK1 interplays with transcription factor GsERF7 from wild soybean to regulate soybean stress resistance. Plant, Cell & Environment **43**, 1192-1211.

Fragoso S, Espindola L, Paez-Valencia J, Gamboa A, Camacho Y, Martinez-Barajas E, Coello P. 2009. SnRK1 isoforms AKIN10 and AKIN11 are differentially regulated in Arabidopsis plants under phosphate starvation. Plant Physiology **149**, 1906-1916.

Gissot L, Polge C, Jossier M, Girin T, Bouly JP, Kreis M, Thomas M. 2006. AKINbetagamma contributes to SnRK1 heterotrimeric complexes and interacts with two proteins implicated in plant pathogen resistance through its KIS/GBD sequence. Plant Physiology **142**, 931-944.

Guerinier T, Millan L, Crozet P, Oury C, Rey F, Valot B, Mathieu C, Vidal J, Hodges M, Thomas M, Glab N. 2013. Phosphorylation of p27(KIP1) homologs KRP6 and 7 by SNF1-related protein kinase-1 links plant energy homeostasis and cell proliferation. Plant Journal **75**, 515-525.

Gutierrez-Beltran E, Elander PH, Dalman K, Dayhoff GW, 2nd, Moschou PN, Uversky VN, Crespo JL, Bozhkov PV. 2021. Tudor staphylococcal nuclease is a docking platform for stress granule components and is essential for SnRK1 activation in Arabidopsis. EMBO Journal **40**, e105043.

Hamasaki H, Kurihara Y, Kuromori T, Kusano H, Nagata N, Yamamoto YY, Shimada H, Matsui M. 2019. SnRK1 Kinase and the NAC Transcription Factor SOG1 Are Components of a Novel Signaling Pathway Mediating the Low Energy Response Triggered by ATP Depletion. Frontiers in Plant Science **10**, 503.

Han C, Liu Y, Shi W, Qiao Y, Wang L, Tian Y, Fan M, Deng Z, Lau OS, De Jaeger G, Bai MY. 2020. KIN10 promotes stomatal development through stabilization of the SPEECHLESS transcription factor. Nature Communications **11**, 4214.

Huang X, Zheng C, Liu F, Yang C, Zheng P, Lu X, Tian J, Chung T, Otegui MS, Xiao S, Gao C, Vierstra RD, Li F. 2019. Genetic Analyses of the Arabidopsis ATG1 Kinase Complex Reveal Both Kinase-Dependent and Independent Autophagic Routes during Fixed-Carbon Starvation. Plant Cell **31**, 2973-2995.

Jamsheer KM, Jindal S, Sharma M, Awasthi P, S S, Sharma M, Mannully CT, Laxmi A. 2022. A negative feedback loop of TOR signaling balances growth and stress-response trade-offs in plants. Cell Reports **39**, 110631.

Jamsheer KM, Sharma M, Singh D, Mannully CT, Jindal S, Shukla BN, Laxmi A. 2018a. FCS-like zinc finger 6 and 10 repress SnRK1 signalling in Arabidopsis. Plant Journal **94**, 232-245.

Jamsheer KM, Shukla BN, Jindal S, Gopan N, Mannully CT, Laxmi A. 2018b. The FCS-like zinc finger scaffold of the kinase SnRK1 is formed by the coordinated actions of the FLZ domain and intrinsically disordered regions. Journal of Biological Chemistry **293**, 13134-13150.

Jeong EY, Seo PJ, Woo JC, Park CM. 2015. AKIN10 delays flowering by inactivating IDD8 transcription factor through protein phosphorylation in Arabidopsis. BMC Plant Biology **15**, 110.

Mair A, Pedrotti L, Wurzinger B, Anrather D, Simeunovic A, Weiste C, Valerio C, Dietrich K, Kirchler T, Nagele T, Vicente Carbajosa J, Hanson J, Baena-Gonzalez E, Chaban C, Weckwerth W, Droge-Laser W, Teige M. 2015. SnRK1-triggered switch of bZIP63 dimerization mediates the low-energy response in plants. Elife **4**.

Ng S, Giraud E, Duncan O, Law SR, Wang Y, Xu L, Narsai R, Carrie C, Walker H, Day DA, Blanco NE, Strand A, Whelan J, Ivanova A. 2013. Cyclin-dependent kinase E1 (CDKE1) provides a cellular switch in plants between growth and stress responses. Journal of Biological Chemistry **288**, 3449-3459.

Nietzsche M, Guerra T, Alseekh S, Wiermer M, Sonnewald S, Fernie AR, Bornke F. 2018. STOREKEEPER RELATED1/G-Element Binding Protein (STKR1) Interacts with Protein Kinase SnRK1. Plant Physiology **176**, 1773-1792.

Nietzsche M, Schiessl I, Bornke F. 2014. The complex becomes more complex: protein-protein interactions of SnRK1 with DUF581 family proteins provide a framework for cell- and stimulus type-specific SnRK1 signaling in plants. Frontiers in Plant Science **5**, 54.

Nukarinen E, Nagele T, Pedrotti L, Wurzinger B, Mair A, Landgraf R, Bornke F, Hanson J, Teige M, Baena-Gonzalez E, Droge-Laser W, Weckwerth W. 2016. Quantitative phosphoproteomics reveals the role of the AMPK plant ortholog SnRK1 as a metabolic master regulator under energy deprivation. Science Reports **6**, 31697.

O'Brien M, Kaplan-Levy RN, Quon T, Sappl PG, Smyth DR. 2015. PETAL LOSS, a trihelix transcription factor that represses growth in Arabidopsis thaliana, binds the energy-sensing SnRK1 kinase AKIN10. Journal of Experimental Botany **66**, 2475-2485.

Pierre M, Traverso JA, Boisson B, Domenichini S, Bouchez D, Giglione C, Meinnel T. 2007. N-myristoylation regulates the SnRK1 pathway in Arabidopsis. Plant Cell **19**, 2804-2821.

Ramon M, Dang TVT, Broeckx T, Hulsmans S, Crepin N, Sheen J, Rolland F. 2019. Default Activation and Nuclear Translocation of the Plant Cellular Energy Sensor SnRK1 Regulate Metabolic Stress Responses and Development. Plant Cell **31**, 1614-1632.

Ruiz-Gayosso A, Rodriguez-Sotres R, Martinez-Barajas E, Coello P. 2018. A role for the carbohydrate-binding module (CBM) in regulatory SnRK1 subunits: the effect of maltose on SnRK1 activity. Plant Journal **96**, 163-175.

Song J, Shang L, Wang X, Xing Y, Xu W, Zhang Y, Wang T, Li H, Zhang J, Ye Z. 2021. MAPK11 regulates seed germination and ABA signaling in tomato by phosphorylating SnRKs. Journal of Experimental Botany **72**, 1677-1690.

Song Y, Zhang H, You H, Liu Y, Chen C, Feng X, Yu X, Wu S, Wang L, Zhong S, Li Q, Zhu Y, Ding X. 2019. Identification of novel interactors and potential phosphorylation substrates of GsSnRK1 from wild soybean (Glycine soja). Plant, Cell & Environment **42**, 145-157.

Sun D, Fang X, Xiao C, Ma Z, Huang X, Su J, Li J, Wang J, Wang S, Luan S, He K. 2021. Kinase SnRK1.1 regulates nitrate channel SLAH3 engaged in nitrate-dependent alleviation of ammonium toxicity. Plant Physiology **186**, 731-749.

Tsai AY, Gazzarrini S. 2012. AKIN10 and FUSCA3 interact to control lateral organ development and phase transitions in Arabidopsis. Plant Journal **69**, 809-821.

Wang W, Lu Y, Li J, Zhang X, Hu F, Zhao Y, Zhou DX. 2021. SnRK1 stimulates the histone H3K27me3 demethylase JMJ705 to regulate a transcriptional switch to control energy homeostasis. Plant Cell **33**, 3721-3742.

Wang Y, Wang L, Micallef BJ, Tetlow IJ, Mullen RT, Feil R, Lunn JE, Emes MJ. 2020. AKINbeta1, a subunit of SnRK1, regulates organic acid metabolism and acts as a global modulator of genes involved in carbon, lipid, and nitrogen metabolism. Journal of Experimental Botany **71**, 1010-1028.

Williams SP, Rangarajan P, Donahue JL, Hess JE, Gillaspy GE. 2014. Regulation of Sucrose non-Fermenting Related Kinase 1 genes in Arabidopsis thaliana. Frontiers in Plant Science **5**, 324.

Xiao S, Jiang L, Wang C, Ow DW. 2020. SnRK1 regulates chromatin-associated OXS3 family proteins localization through phosphorylation in Arabidopsis thaliana. Biochemical and Biophysical Research Communications **533**, 526-532.

Zhai Z, Liu H, Shanklin J. 2017. Phosphorylation of WRINKLED1 by KIN10 Results in Its Proteasomal Degradation, Providing a Link between Energy Homeostasis and Lipid Biosynthesis. Plant Cell **29**, 871-889.
